# Overexpression of a Cotton Gene That Encodes a Putative Transcription Factor of AP2/EREBP Family in *Arabidopsis* Affects Growth and Development of Transgenic Plants

**DOI:** 10.1371/journal.pone.0078635

**Published:** 2013-10-23

**Authors:** Ying Zhou, Hui Xia, Xiao-Jie Li, Rong Hu, Yun Chen, Xue-Bao Li

**Affiliations:** Hubei Key Laboratory of Genetic Regulation and Integrative Biology, College of Life Sciences, Central China Normal University, Wuhan, China; Wuhan University, China

## Abstract

In the study, a gene encoding a putative ethylene response factor of AP2/EREBP family was isolated from cotton (*Gossypium hirsutum*) and designated as *GhERF12*. Sequence alignment showed that GhERF12 protein contains a central AP2/ERF domain (58 amino acids) with two functional conserved amino acid residues (ala14 and asp19). Transactivation assay indicated that GhERF12 displayed strong transcription activation activity in yeast cells, suggesting that this protein may be a transcriptional activator in cotton. Quantitative RT-PCR analysis showed that *GhERF12* expression in cotton was induced by ACC and IAA. Overexpression of *GhERF12* in *Arabidopsis* affected seedling growth and development. The *GhERF12* transgenic plants grew slowly, and displayed a dwarf phenotype. The mean bolting time of the transgenic plants was delayed for about 10 days, compared with that of wild type. Further study revealed that some ethylene-related and auxin-related genes were dramatically up-regulated in the transgenic plants, compared with those of wild type. Collectively, we speculated that GhERF12, as a transcription factor, may be involved in regulation of plant growth and development by activating the constitutive ethylene response likely related to auxin biosynthesis and/or signaling.

## Introduction

AP2/EREBP (APETALA2/ethylene responsive element binding protein) family is one of the largest families of plant transcription factors and plays important roles in plant growth and development [1]. AP2/EREBP protein at least contains one highly conserved AP2/EREBP DNA-binding domain consisting of about 60 amino acids [2]. According to the number of the AP2/EREBP DNA-binding domain, this family can be classified into two subfamilies. One is AP2 subfamily, of which the protein contains two AP2/EREBP DNA-binding domains, and the other is EREBP subfamily, of which the member has only one AP2/EREBP DNA-binding domain. The EREBP subfamily is further divided into three subgroups: RAV (Related to ABI3/VP1), DREBP (dehydration-responsive element binding protein) and ERF (ethylene response factor) [3,4]. RAV transcription regulators have a B3 DNA binding domain following one AP2 domain. The member of DREB and ERF subgroups contains one single AP2 domain in its sequence, but the divergence of two conserved amino acids is supposed to explain the functional difference between the two subgroups. Amino acid residues 14 and 19 in AP2 domain of DREB protein are V (Val_14_) and E (Glu_19_), in contrast to A (Ala_14_) and D (Asp_19_) in ERF protein [3]. 

Ethylene (C_2_H_4_) is the chemically simplest plant hormone. Among ethylene-induced reactions of plants, triple response in the etiolated seedlings is the most prominent phenotype that show bended cotyledons and inhibited cell elongation of hypocotyls and roots in dark [5]. In the course of the response, ERF is a downstream component in the ethylene signaling pathway to regulate the plant development [6]. It was reported that ERF1 acts downstream of EIN3 in the ethylene signaling pathway and constitutive expression of ERF1 results in the activation of a variety of ethylene response genes in *Arabidopsis* [7]. Overexpression of *OsERF1* in *Arabidopsis* activated a variety of ethylene response genes and resulted in a similar phenomenon to the characterized AtERF1 [8].

Auxin (indole-3-acetic acid, IAA) plays a central role in plant growth and development, including cell division, expansion and differentiation, patterning of embryos, vasculature and other tissues, and distribution of growth between root and shoot meristems [9]. Data obtained so far for AP2/EREBP proteins in plants (such as *Arabidopsis*, petunia, maize, rice and tobacco) suggest that members of the AP2 subfamily play roles in auxin signaling [10]. MtPLETHORA1 and MtPLETHORA2 are strongly induced by auxin addition and play a role in the auxin-induced root formation [11]. *Arabidopsis PUCHI*, which encodes a putative APETALA2/ethylene-responsive element binding protein transcription factor, is required for cell divisions during lateral root formation. PUCHI, acting downstream of auxin signaling, is induced by exogenous auxin and regulated by ARF proteins that are activated by auxin during early lateral root primordium development [12]. CRL5, a member of AP2/ERF transcription factor family, functions in downstream of AUX/IAA and ARF-mediated auxin signaling pathway involved in crown root initiation [13].

Auxin-ethylene cross-talk is known to interact in the regulation of several biological processes, such as root elongation, differential growth of hypocotyls, and root hair formation [14]. It has been reported that the effects of ethylene on root growth is largely mediated by auxin biosynthesis and transport-dependent local auxin distribution [15]. Rahman et al. (2001) demonstrated that exogenously applied auxin can recover the ethylene response in *aux1* and *eir1* mutants [16]. The further increase in auxin levels in responsive tissues by application of high ethylene concentrations elicits inhibition of root growth in *eir1* mutant [15]. Furthermore, previous study revealed that ethylene may regulate auxin biosynthesis and transport from the root apex to elongation zone tissues for enhancing inhibition of root growth [17].

Cotton (*Gossypium hirsutum*), which produces natural textile fibers and cottonseed oils, is an important crop in the world. Cotton development is a complicated and ordered process regulated by a large amount of genes. In this study, we identified a *GhERF12* gene, which encodes a putative transcription factor of the AP2/EREBP family, in cotton. Quantitative RT-PCR analysis showed that *GhERF12* expression in cotton was induced by ACC and IAA. Overexpression of *GhERF12* in *Arabidopsis* affected seedling growth and development. Expressions of some ethylene-related and auxin-related genes were altered in the transgenic plants, suggesting that GhERF12 may be involved in regulation of plant growth and development by modulating ethylene as well as auxin signaling in cotton.

## Materials and Methods

### Cotton materials

Cotton (*Gossypium hirsutum* cv. Coker312) seeds were surface-sterilized with 70% ethanol for 60 sec and 10% H_2_O_2_ for 90 min, followed by washing with sterile water. The sterilized seeds were germinated on half-strength MS medium under a 16 h light/8 h dark cycle at 28°C for 5 days. Roots, cotyledons and hypocotyls were cut from these sterile seedlings. The other organs/tissues (such as leaves, petals, anthers, ovules and fibers) were derived from cotton plants grown in field for isolating total RNA.

For ACC (ethylene precursor 1-aminocyclopropane-1-carboxylic acid) treatment, 5-day-old cotton seedlings were placed in half-strength MS liquid medium with 200 μM ACC for 2, 4, 6, 8 and 12 h. Five-day-old cotton seedlings were also cultured in half-strength MS liquid medium without any supplements as control. Roots of the treated seedlings and control were collected for further analysis.

For IAA (indole acetic acid) treatment, five-day-old cotton seedlings were placed in half-strength MS liquid medium with 100 μM IAA for 2, 4, 6 h, using five-day-old cotton seedlings cultured in half-strength MS liquid medium without any supplements as control. Roots of the treated seedlings and control were collected for further analysis.

### Isolation of *GhERF12* cDNA

Over 4,000 cDNA clones were randomly selected from cotton seedling cDNA library for sequencing. One cDNA clone encoding an AP2 domain protein (designated as *GhERF12*) was identified for further characterization. 

### Protein sequence and phylogenetic analysis

DNA and protein sequences were analyzed by DNASTAR software (DNAStar Inc., Madison, WI, USA). The conserved domain was confirmed at NCBI (http://blast. ncbi.nlm.nih.gov/Blast.cgi). Sequence alignment and protein motif analysis were performed with ClustalW (http://www.ebi.ac.uk/clustalw/). Protein sequences were aligned with the ClustalX program (http://bips.u-strasbg.fr/fr/Documentation/ClustalX/).

### Transactivation activity assay

To investigate the transcriptional activity of GhERF12, the coding sequence of *GhERF12* was amplified by PCR using the proofreading *Pfu* DNA polymerase and gene-specific primers, and was cloned into the *Eco*R I and *Pst* I restriction sites of pGBKT7 (Biosciences Clontech, Palo Alto, CA, USA) which contains the GAL4 DNA binding domain to create the fusion construct of pGBKT7-GhERF12. The construct was transformed into the yeast strain AH109 and Y187, and two reporter genes *ADE2* and *lacZ* were tested by streaking the yeast AH109 transformants on SD/-Trp/-Ade medium (SD minimal medium lacking Trp and Ade) (Clontech Inc., Palo Alto, CA,USA) and the flash-freezing filter assay of yeast Y187 transformants, respectively (James et al., 1996). The yeast harboring pGBKT7 vector was used as the negative control. The yeast harboring pGBKT7-53 which encoded a fusion of GAL4 DNA-BD/murine p53 and pGADT7-RecT which encoded a fusion of the GAL4 DNA-AD/SV40 large T-antigen was used as the positive control. 

### Construction of *GhERF12* expression vector

The open-reading frame (ORF) of *GhERF12* gene was amplified by PCR, using the proofreading *Pfu* DNA polymerase and gene-specific primers P1: 5’-cttcccgggatggaagaacccactttattc-3’ and P2: 5’-cttctcgagctaccaaggaacattagtctc-3’, which contained added *Sma*I and *Xho*I enzyme sites, respectively. After digestion, the amplicon was inserted into the tool vector pMD to construct *GhERF12*-overexpression vector under the control of CaMV 35S promoter.

### *Arabidopsis* transformation and plant growth conditions

The *Arabidopsis thaliana* Columbia ecotype was used for this study. The construct was introduced into *Arabidopsis* by the floral dip method. Positive transformants were selected on MS medium with 50 mg/L kanamycin and grew until maturation. The homozygous *GhERF12*-overexpression transgenic lines of T3 and T4 generations were used for phenotypic analysis.

For IAA treatment, seeds of transgenic *Arabidopsis* and wild type were germinated and grew on MS medium containing 0 (control), 10, 20, 30, 50, 100 nM IAA. After the seeds were stratified at 4 °C for 2 days, the MS agar plates with the germinated seeds were placed vertically in an incubator with a photoperiod of 16-h light/8-h dark at 22 °C. The length of the primary roots and lateral root number of the seedlings were measured at 8th day after germination (n = 60 - 100). 

For ACC treatment, seeds of transgenic *Arabidopsis* and wild type were germinated and grew on MS medium with 0.5 μM ACC or without ACC (control). Length of primary roots and hypocotyls of the seedlings was measured at 3rd day after germination (n=60 to 100). 

### Quantitative RT-PCR analysis

Total RNA was extracted and purified using the Qiagen RNeasy mini kit (Qiagen, Hilden, Germany). First-strand of cDNA was reversely synthesized from the RNA using M-MLV reverse transcriptase (Promega, Madison, WI, USA) according to the manufacturer’s instructions. *GhERF12* expression in cotton fibers were analyzed by quantitative PCR using the fluorescent intercalating dye SYBR-Green in a detection system (Opticon2; MJ Research) as described previously [18]. A cotton polyubiquitin gene (*GhUBI1*, access number in GenBank: EU604080) was used as a standard control in RT-PCR reactions. The specific *GhERF12* primers used in RT-PCR were: 5’-CCCTGTTGTGGCTCTTAAAAAG-3’ and 5’- CTAAAATTCACCCTCCATCAAC -3’. *GhUBI1* primers used in RT-PCR were: 5’-CTGAATCTTCGCTTTCACGTTATC-3’ and 5’-GGGATGCAAATCTTCGTGAAAAC-3’. 

The expression of *GhERF12* gene in the transgenic *Arabidopsis* plants was analyzed by quantitative reverse transcriptase (RT)-PCR as described earlier [18], and using the *ACTIN2* gene as a quantitative control. To assay the expression of ethylene-regulated genes and auxin-response genes in the transgenic *Arabidopsis* plants, RT-PCR analysis was performed with the RNA samples isolated from 8-day-old seedlings. All the quantitative RT-PCR experiments were repeated three times. Primers used in RT-PCR to check *GhERF12* expression in the transgenic lines were described as above. The primers of *Arabidopsis* genes used in RT-PCR were: *b-chi*, 5’-TTCTGGATGACTGCTCAGCC-3’ and 5’-GAGGCCGTTAACGAAGGATC-3’; *PDF1.2*, 5’-CATGGCTAAGTTTGCTTCCATC-3’ and 5’-CATGGGACGTAACAGATACAC-3’; *SAUR*, 5’-TATTGTTAAGCCGCCCATTG-3’ and 5’-AAGGGAATCATCGTCGACAC-3’; *IAA9*, 5’-GAGCTGCTGGGAAGGATATG-3’ and 5’-GCTGCAGCTAACCCAATAGC-3’; *IAA17*, 5’-AGGGTTCTCAGAGACGGTTG-3’ and 5’-TTGATTTTTGGCAGGAAACC-3’; *IAA19*, 5’-GACTCGGGCTTGAGATAACG-3’ and 5’-CGTGGTCGAAGCTTCCTTAC-3’; *HAT2*, 5’-CCACCAACTACACTCATCATG-3’ and 5’-TCATGAGAAGGCCAATCATCC-3’; *GH3.2*, 5’-TGCGTGAGCTTCACACCTATC-3’ and 5’-GGATTCCAACAGAAGATGAAGG-3’; *Pin1*, 5’-CCTGGCGCAGACAGAAATGAC-3’ and 5’-ACAAGTACAGGGCTAGATGGC-3’.

## Results

### Isolation and characterization of *GhERF12* gene

**Figure 1:**
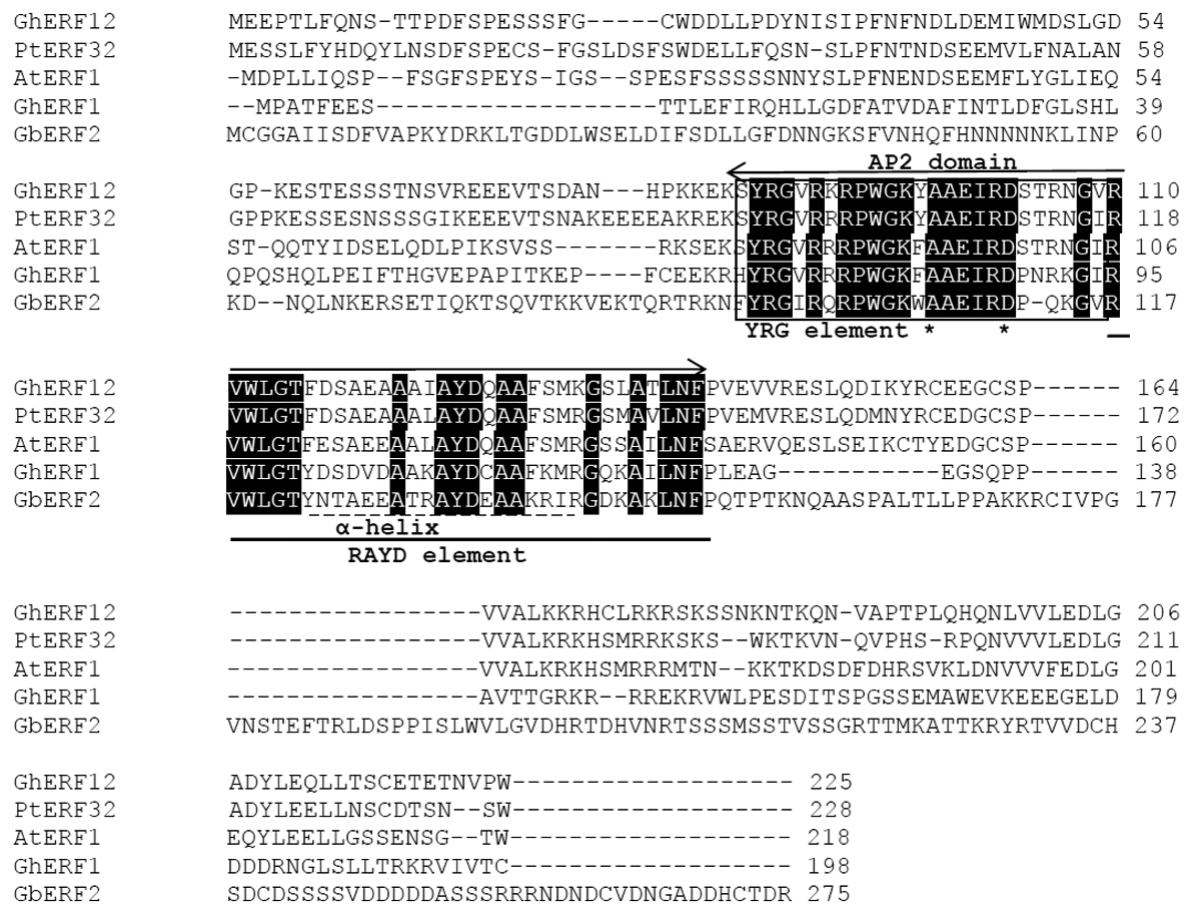
Sequence alignment of GhERF12 and the other plant AP2/EREBP proteins. The proteins and their accession numbers used for alignment are listed below: PtERF32 (XP_002306842.1), AtERF1 (NP_188965.1), GhERF1 (AAO59439), GbERF2 (AAT77191). Arrow indicates a conserved AP2/EREBP DNA-binding domain; Dotted line shows the α-helix; Black box region shows YRG element; Asterisks (*) indicate that amino acids 14th and 19th in the AP2 domain were A (Ala) and D (Asp); Line shows RAYD element.

We isolated the full-length cDNA sequence of *GhERF12* from cotton cDNA library (accession number in GenBank: KF430217)*. GhERF12* cDNA is 905bp in length, and includes 678bp of open reading frame (ORF) encoding an ERF protein of 225 amino acids. GhERF12 protein shares relatively high similarities to *Populus trichocarpa* PtERF32 (XP_002306842), *Arabidopsis* AtERF1 (AAM63284), cotton (*Gossypium hirsutum*) GhERF1 (AAO59439), and cotton (*Gossypium barbadense*) GbERF2 (AAT77191) proteins. It contains a single AP2/EREBP DNA-binding domain (From K_84_ to F_142_). The AP2 domain, which aligned to the length of four AP2 members, contains two conserved sequence elements. The first element is YRG element consisting of 25 amino acids with the conserved YRG motif. 14th and 19th amino acids in AP2 domain are A (Ala) and D (Asp) in the YRG element. The second element is RAYD element that is 33 amino acids in length and contains a highly conserved 18 amino acid core region predicting to form an amphipathic a-helix in the AP2 domains. As expected, AP2 domain is the area of the highest conservation in GhERF12 protein (Figure 1). 

### GhERF12 expression is induced by ACC and IAA

**Figure 2:**
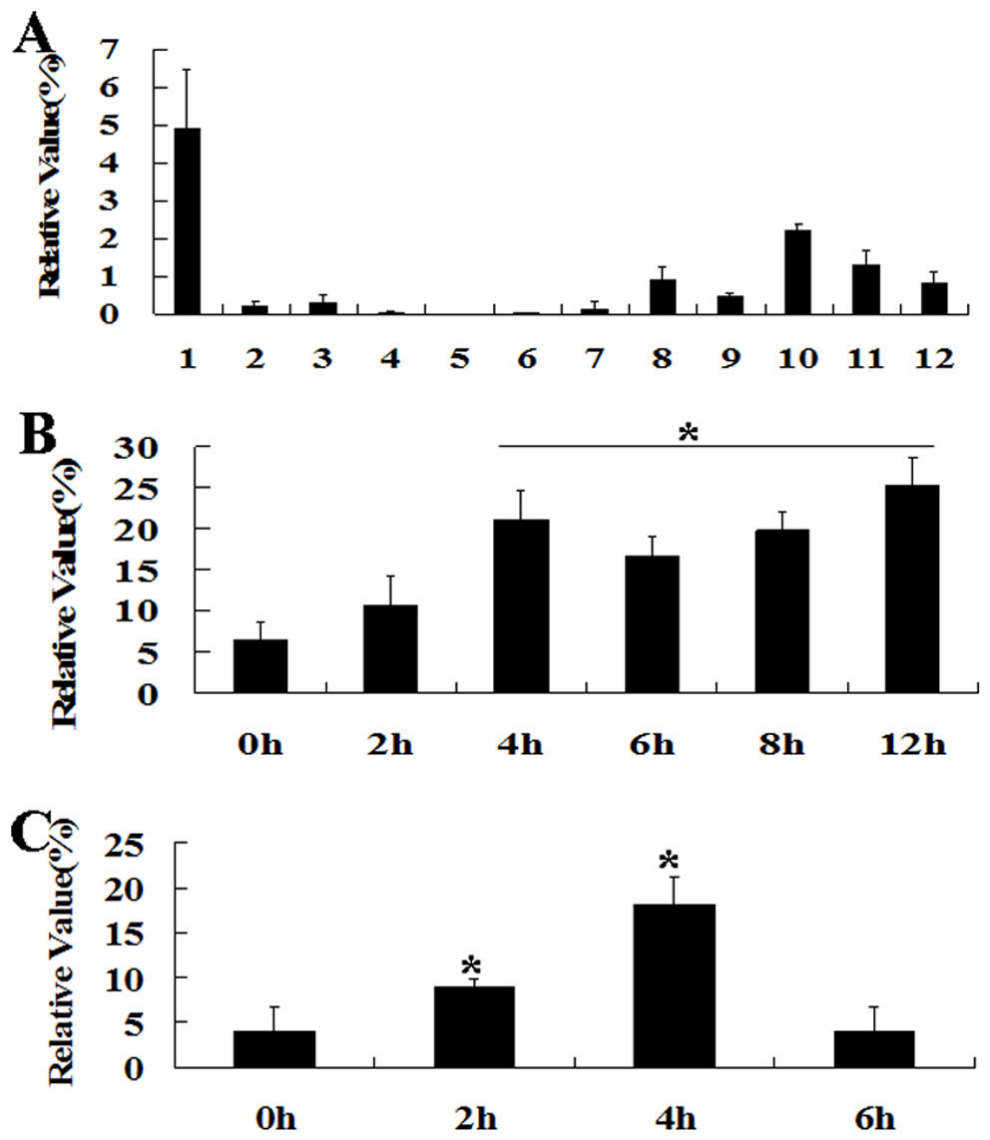
Quantitative RT-PCR analysis of *GhERF12* expression in cotton tissues and in response ACC and IAA treatments. (**A**) Expression profiling of *GhERF12* gene in cotton tissues. (**B**) Analysis of *GhERF12* expression in roots of the five-day-old cotton seedlings treated with 200 μM ACC (1-aminocyclopropane-D-carboxylic acid) for 0, 2, 4, 6, 8 and 12 h. (**C**) Analysis of *GhERF12* expression in roots of the five-day-old cotton seedlings treated with 100 μM IAA for 0, 2, 4, 6 h. Total RNA was isolated from cotton tissues: 1, roots; 2, hypocotyls; 3, cotyledons; 4, leaves; 5, petals; 6, anthers; 7, ovules; 8, 3 DPA fibers; 9, 6 DPA fibers; 10, 9 DPA fibers; 11, 12 DPA fibers; 12, 15 DPA fibers. Relative values of *GhERF12* expression in cotton tissues are shown as a percentage of *GhUBI1* expression activity. Independent *t*-tests demonstrated that there was significant difference (* P < 0.05) in *GhERF12* expression between the treated seedlings and controls. DPA, day post anthesis.

To investigate the expression profiling of *GhERF12* in cotton, RT-PCR technique was employed to analyze *GhERF12* expression pattern in cotton tissues. As shown in Figure 2A, *GhERF12* was preferentially expressed in roots, and at relatively high level in the developing fibers, but its transcripts was detected at relatively low levels in the other tissues (such as petals, anthers, leaves, hypocotyls, ovules and cotyledons) of cotton. During fiber development, *GhERF12* transcripts were accumulated at the highest level in 9 DPA (day post anthesis) fiber cells.

 To study the effects of ACC (1-aminocyclopropane-D-carboxylic acid) on *GhERF12* expression, five-day-old seedlings were treated with ACC and then total RNAs were isolated from the roots of these treated seedlings and controls for quantitative RT-PCR analysis. The results showed that expression of *GhERF12* was remarkably up-regulated in seedlings cultured in half-strength MS medium with ACC. As shown in Figure 2B, expression level of *GhERF12* was slightly enhanced in roots of the seedlings under ACC treatment for 2 h, and then remarkably increased in roots of the seedlings treated with ACC for 4 - 12 h, suggesting that *GhERF12* may be involved in response to ethylene signaling during root development of cotton.

 To study the effects of IAA (indole acetic acid) on *GhERF12* expression, five-day-old seedlings were treated with IAA and then total RNAs were isolated from the roots of these treated seedlings and controls for quantitative RT-PCR analysis. The results showed that *GhERF12* expression was remarkably up-regulated in seedlings cultured in half-strength MS medium with IAA. As shown in Figure 2C, expression level of GhERF12 was remarkably enhanced in roots of the seedlings under IAA treatment for 2 h, and reached to its peak value in roots after 4 h of IAA treatment. However, its signals were declined to normal level at 6 h after IAA treatment. These results suggested that GhERF12 may also be involved in response to auxin signaling during root development of cotton.

### Transactivation assay of GhERF12 protein

**Figure 3:**
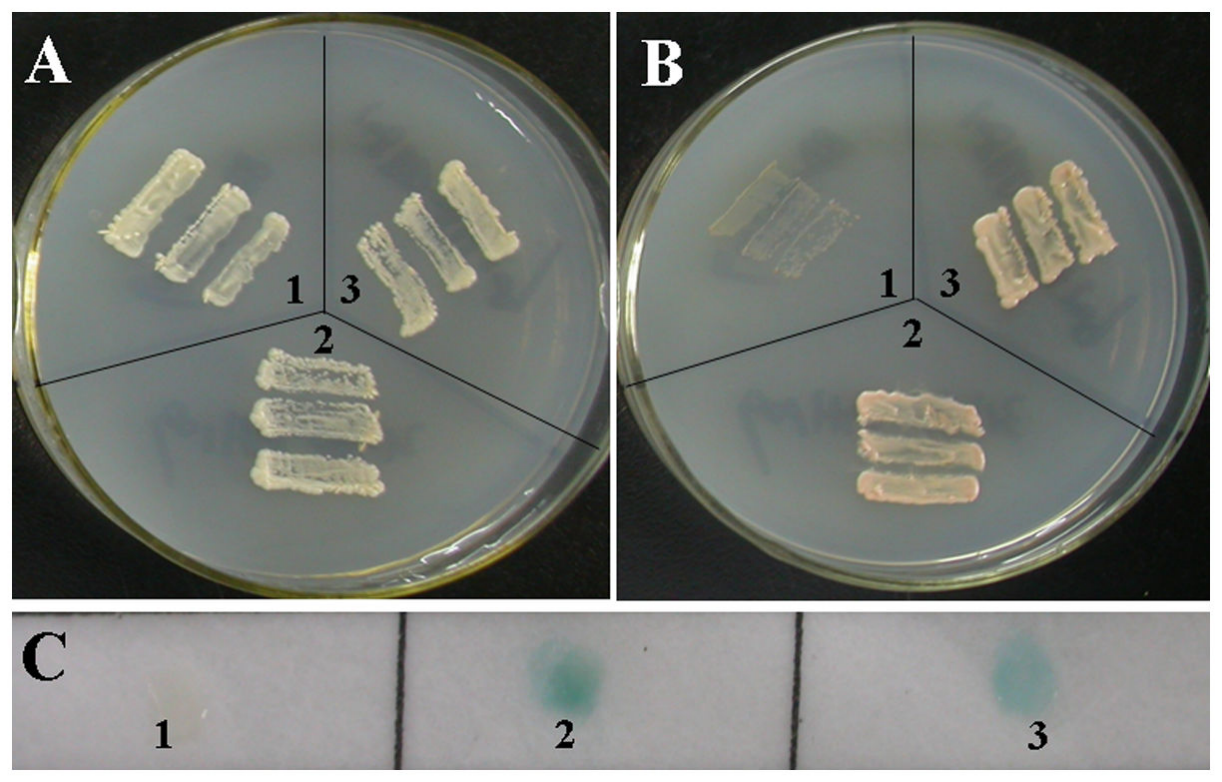
Transactivation activity assay of GhERF12 protein in yeast cells. (**A**) Yeast transformants were streaked on SD/-Trp medium (SD minimal medium lacking Trp); (**B**) Yeast transformants were streaked on SD/-Trp/-Ade medium (SD minimal medium lacking Trp and Ade); (**C**) flash-freezing filter assay of the β-galactosidase activity. 1, Yeast transformants of pGBKT7 was used as the negative control; 2, Yeast transformants of pGBKT7-53/pGADT7-RecT was used as the positive control; 3, Yeast transformants of pGBKT7-GhERF12.

To investigate the transcriptional activation activity of GhERF12, we performed a transient expression assay using a GAL4-responsive reporter system. The effector plasmid contained the GAL4 binding domain coding region and GhERF12 was transferred into yeast strain AH109. We found that transformed yeast cells including pGBKT7-GhERF12 could grow successfully on SD/-Trp medium (SD minimal medium lacking Trp) and SD/-Trp/-Ade medium (SD minimal medium lacking Trp and Ade). We also transferred the effector plasmid into the yeast strain Y187 harboring reporter gene *LacZ* and used flash-freezing filter assay. The yeast colonies including pGBKT7-GhERF12 turned blue, showing positive β-galactosidase activity, like the yeast cells containing pGBKT7-53 and pGADT7-RecT (positive control). On the contrary, yeast cells harboring only the pGBKT7 vector (negative control) could not grow on SD/-Trp/-Ade plate and could not turn blue in flash-freezing filter assay (Figure 3). The above results suggested that GhERF12 protein may be an active transcriptional activator in cotton.

### Overexpression of *GhERF12* in *Arabidopsis* hinders plant growth by regulating the genes related to ethylene response

**Figure 4:**
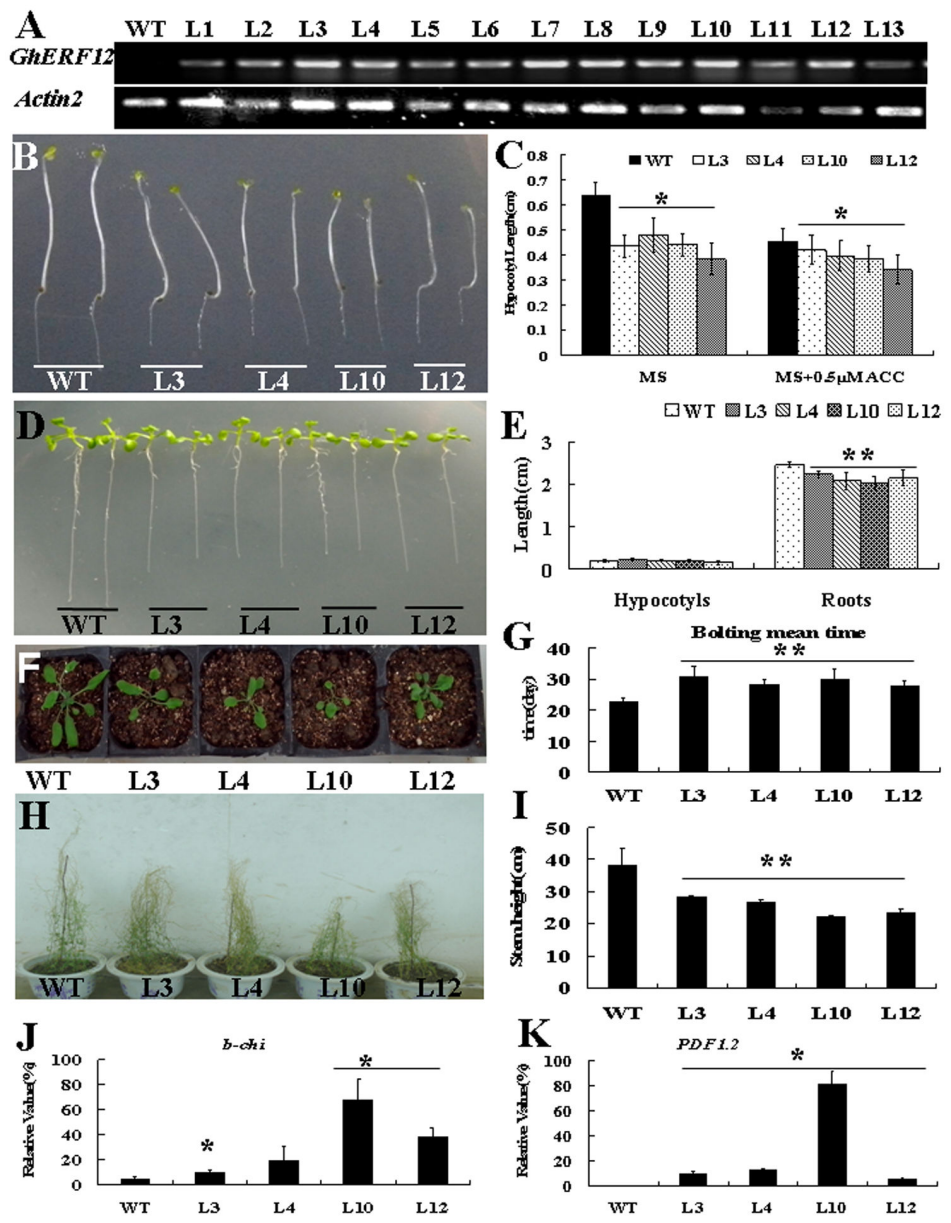
Phenotypic assay of the *35S*:*GhERF12*-overexpression transgenic *Arabidopsis* plants. (**A**) RT-PCR analysis of *GhERF12* expression in transgenic *Arabidopsis* plants (lines L1 – L13). (**B**) *GhERF12* transgenic and wild type seedlings grown on MS medium at 22 °C in dark for 3 days. (**C**) Measurement and statistic analysis of hypocotyl length of three-day-old etiolated wild type and *GhERF12* transgenic seedlings grown on MS medium with or without 0.5 μM ACC (1-aminocyclopropane-D-carboxylic acid) in dark for 3 days. (**D**) *GhERF12* transgenic and wild type seedlings grown on MS medium at 22 °C under a 16 h light/8 h dark cycle for 8 days. (**E**) Measurement and statistic analysis of root length of eight-day-old *GhERF12* transgenic and wild type seedlings grown on MS medium under a 16 h light/8 h dark cycle for 8 days. (**F**) Morphology of wild type and *GhERF12*-overexpression transgenic plants at vegetative growth stage. (**G**) A diagram of mean bolting time of wild type and *GhERF12*-overexpression transgenic plants at vegetative growth stage. (**H**) Comparison of stem height between mature *GhERF12* transgenic plants and wild type grown in culture room (22°C, 16h light/8h dark) (**I**) Measurement and statistic analysis of stem height of the transgenic lines and wild type. (**J**) Quantitative RT-PCR examination of the transcripts of ethylene-responsive gene *b-chi* (*basic-chitinase*) in wild type and *GhERF12* transgenic plants. (**K**) Quantitative RT-PCR examination of the transcripts of ethylene-responsive gene *PDF1.2* in wild type and *GhERF12* transgenic plants. Total RNA was prepared from roots of 8-day-old wild type and *GhERF12* transgenic seedlings. Independent *t*-tests demonstrated that there was significant (*P < 0.05) or very significant (**P < 0.01) difference between the transgenic lines and wild type. WT, wild type; L1 – L13, *GhERF12*-overexpression transgenic *Arabidopsis* lines.

To investigate the role of GhERF12 in the ethylene signaling pathway, *GhERF12* overexpression construct was introduced into *Arabidopsis* to generate the transgenic plants constitutively expressing GhERF12 under the control of CaMV 35S promoter. Thirteen homozygous lines of the T3 generation were selected for further analysis. The expression levels of *GhERF12* in these transgenic progeny plants were examined by RT-PCR analysis using gene-specific primers. As shown in Figure 4A, *GhERF12* expression was detected in the transgenic lines (L1 – L13) displayed, but *GhERF12* transcripts were not detected in wild type. When the seedlings grew in dark for three days, hypocotyls of the *GhERF12* transgenic *Arabidopsis* seedlings were shorter than those of wild type (Figure 4B). Measurement and statistical analysis indicated that there was significant difference in the hypocotyl length between the *GhERF12* transgenic seedlings and wild type. Under ACC treatment, however, both the transgenic and wild type seedlings in dark showed the phenotype of inhibition of hypocotyl elongation (Figure 4C). We further examined root growth of wild type and transgenic seedlings cultured on MS medium under light. When seedlings were cultured under light, primary roots of the transgenic seedlings grew more slowly than those of wild type (Figure 4D). Measurement and statistical analysis indicated that there was significant difference in length of primary roots between the 8-day-old *GhERF12* transgenic plants and wild type, but no obvious alteration in hypocotyl length of the 8-day-old transgenic seedlings, compared with that of wild type (Figure 4E). These data suggested that overexpression of *GhERF12* in *Arabidopsis* hindered hypocotyl elongation of the transgenic seedlings in dark and primary root growth of the transgenic plants under light. 

As the seedlings were transplanted into soil for further developing, the *GhERF12* transgenic plants grew slowly, and showed a dwarf phenotype, similar to that of the constitutive ethylene response mutant *ctr1* and EIN3/EIL1-, AtERF1- and OsERF1-overexpression transgenic plants. As shown in Figure 4F, the 4-week-old transgenic plants were much smaller than wild type. Compared with that of wild type, the mean bolting time of the transgenic plants was delayed for about 10 days (Figure 4G). When plants nearly matured, the *GhERF12* overexpression transgenic plants were still shorter than wild type (Figure 4H). Measurement and statistical analysis indicated that there was significant difference in stem height between the mature *GhERF12* transgenic plants and wild type. The stem height of *GhERF12* transgenic plants was 10 - 15 cm less than that of wild type (Figure 4I). The results suggested that overexpression of *GhERF12* in *Arabidopsis* affected plant growth and development, and consequently, the transgenic plants displayed a dwarf phenotype.

To confirm whether the defective phenotype of the *GhERF12* transgenic plants is due to activation of ethylene response, the expression of two ethylene-regulated genes, *b-chi* (basic chitinase) and *PDF1.2*, was examined in 8-day-old seedlings grown at 22 °C under a 16 h-light/8 h-dark photoperiod. The results revealed that weak expression of *b-chi* gene was detected in wild type, whereas the greatly enhanced activity of *b-chi* gene was found in the transgenic plants (Figure 4J). Similarly, moderate to strong expression of *PDF1.2* gene was observed in *GhERF12* transgenic plants, but the expression of this gene was undetectable in wild type (Figure 4K). These results suggested that GhERF12 may promote transcription of ethylene-regulated genes and activate the constitutive ethylene response.

### *GhERF12*-overexpression transgenic plants display the altered auxin response

**Figure 5:**
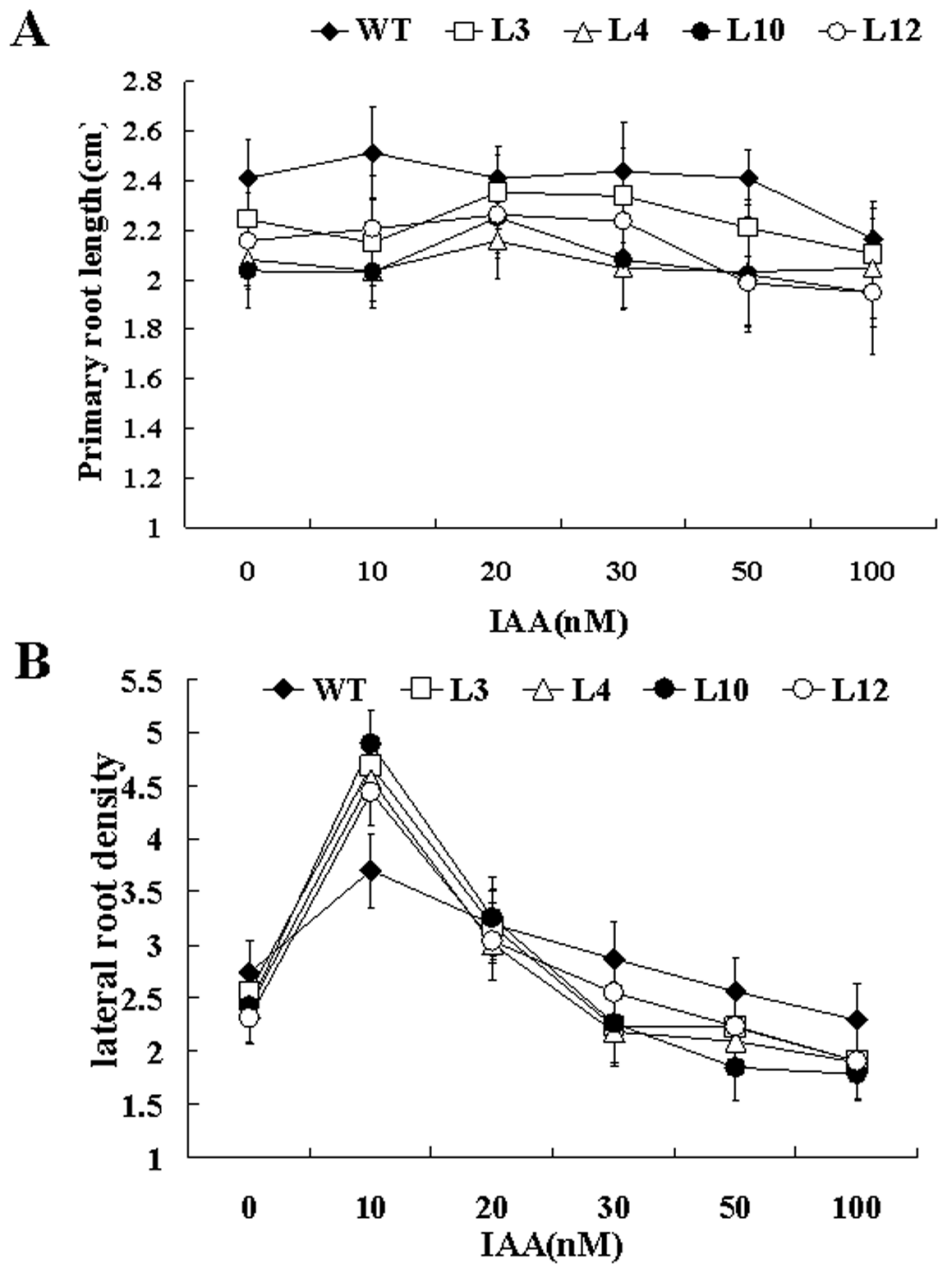
Comparison of primary root length and lateral root density between wild type and *GhERF12* transgenic *Arabidopsis* seedlings on MS medium with different concentrations of IAA. The seedlings were cultured on MS medium without or with various concentrations (10, 20, 30, 50 and 100 nM) of indole acetic acid (IAA) at 22 °C (16 h light/8 h dark) for 8 days. (**A**) Measurement and statistic analysis of primary root length of wild type and *GhERF12* transgenic *Arabidopsis* seedlings. (**B**) Measurement and statistic analysis of lateral root density (number of lateral roots/cm primary root) of wild type and *GhERF12* transgenic *Arabidopsis* seedlings. The experiments were repeated three times, and error bars represent standard deviation. WT, wild type; L3, L4, L10 and L12, four *GhERF12* transgenic lines.

**Figure 6:**
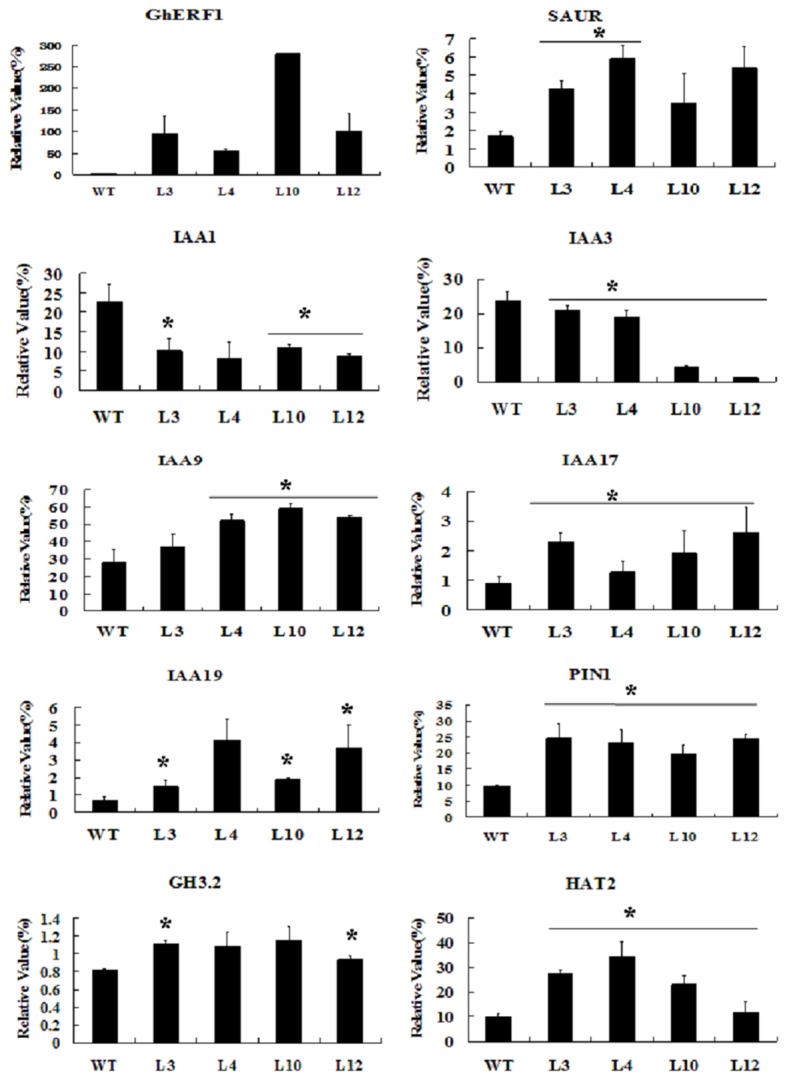
Quantitative RT-PCR analysis of expression of the auxin-related genes in *GhERF12*-overexpression transgenic *Arabidopsis* seedlings. Total RNA was isolated from roots of the 8-day-old transgenic and wild type seedlings grown on MS medium under normal conditions (22 °C, 16 h light/8 h dark). Transcript levels of cotton *GhERF1*, and *Arabidopsis* *SAUR*, *IAA1*, *IAA3*, *IAA9*, *IAA17*, *IAA19*, *HAT2*, *GH3.2* and *PIN1* genes in the seedlings were determined by quantitative RT-PCR, using *Arabidopsis* *Actin2* gene as a quantification control. Independent *t*-tests demonstrated that there was significant difference (P < 0.05) between the transgenic lines and wild type. WT, wild type; L3, L4, L10 and L12, four *GhERF12* transgenic lines.

In addition to ethylene, auxin also plays the important role in roots elongation. We determined the effects of exogenous auxin on root growth of wild type and *GhERF12*-overexpression transgenic lines in light. Seedlings of wild type and the transgenic lines were transferred to MS medium containing various concentrations of auxin after seeds germinated, and length of primary roots and lateral root number of 8-day-old seedlings were measured (n>50). As shown in Figure 5A, the primary roots of the *GhERF12*-overexpression transgenic seedlings grew slower than those of wild type when they were cultured on MS medium with or without IAA (indole acetic acid). On the other hand, when cultured on MS medium without IAA, the lateral root density of the *GhERF12*-overexpression transgenic seedlings was slightly lower than that of wild type. After supplemented with 10 nM IAA, the lateral root density of the transgenic seedlings became much higher than that of wild type. With the increased exogenous IAA concentration, however, the lateral root density of the transgenic seedlings was significantly reduced, compared with that of wild type (Figure 5B). Since the above results suggested that GhERF12 may be involved in the transgenic plant response to auxin signaling during root development, we analyzed expression of genes related to auxin synthesis (such as *HAT2* and *GH3.2*) and signaling pathway (such as *IAA1*, *IAA3*, *IAA9*, *IAA17*, *IAA19*, SMALL AUXIN-UP RNA (SAUR), and *PIN1*) in the *GhERF12* transgenic plants grown on MS medium without the application of exogenous IAA. The results revealed that *GhERF12*-overexpression transgenic *Arabidopsis* plants displayed the altered expression levels of these auxin-related genes (Figure 6). Together, these data suggested that GhERF12 may activate the constitutive ethylene response likely related to auxin biosynthesis and signaling for regulating plant growth and development.

## Discussion

In this study, we identified the *GhERF12* gene which encodes an ERF transcription factor in cotton. It has been indicated that the ERF subgroup is different from the DREB subgroup by two conserved amino acid residues in the AP2/ERF domain. That is, the 14th valine and the 19th glutamic acid are conserved in the DREB proteins, whereas alanine and aspartic acid residues are conserved at the corresponding positions of ERF proteins [3]. In agreement with other ERF proteins, GhERF12 has the 14th alanine and 19th aspartic acid in the AP2/ERF domain, suggesting that GhERF12 is a member of the ERF subfamily. ERF proteins have been shown to act as transcription activators or repressors. Tobacco NtERF3, and *Arabidopsis* AtERF3/4 and AtERF7–12 characterized by the well-defined EAR repressor domain, are active repressors [19–21]. NtERF2/4, AtERF1/2/5, periwinkle ORCA2/3, and tomato Pti4 function as transcription activators [19,20,22–24]. GhERF12 protein has high sequence similarity with AtERF1. GhERF12 was capable of activating transcription of reporter genes in yeast cells, suggesting that GhERF12 acts as a transcription activator to regulate the expression of its target genes in cotton development. 

It has been reported that the members of AP2/EREBP family in plants play important roles in regulation of organ development, cell division and differentiation, etc [25–30]. In cotton, *GhERF4* is constitutively expressed in leaves, roots, seeds and stems, while *GhERF2* and *GhERF3* transcripts are accumulated at higher levels in roots, stems, leaves and seeds, relatively lower levels in embryos, flowers and fibers, and a very low level in cotyledons [31]. High levels of *GhERF6* products were found in leaves, stems and roots, but no or low expression of this gene was found in other tissues of cotton [32]. In this study, our results showed that *GhERF12* was preferentially expressed in roots, and at relatively high expression level in fibers, but at very low levels in other tissues (Figure 2). Previous study revealed that *AtERF1* expression is rapidly induced in plant response to ethylene. *AtERF1* mRNA began to be accumulated in plants after 15 min of ethylene treatment [7]. *OsERF1* mRNA accumulation is obviously increased in rice after ethylene inducement [8]. Similarly, *GhERF12* was significantly up-regulated in cotton roots with ACC treatment, suggesting that *GhERF12* may be involved in plant response to ethylene signaling during root development of cotton.

Through the gene overexpression in *Arabidopsis*, we further investigated the role of *GhERF12* in plant development. The experimental results showed that hypocotyls of *GhERF12* transgenic *Arabidopsis* seedlings in dark were stunted, similar to that of wild type treated with ACC. In light, roots of the transgenic seedlings were remarkably shorter than those of wild type, and the transgenic adult plants still displayed a dwarf phenotype and the delayed mean bolting time (Figure 4). These data suggested that constitutive ethylene response may be activated by overexpression of *GhERF12* in the transgenic *Arabidopsis*. Similar phenotypes have been found in the *AtERF1* and *AtEIN3* transgenic *Arabidopsis* plants [7,33]. Furthermore, the expression of two ethylene-responsive genes (*b-chi* and *PDF1.2*) was up-regulated in *GhERF12*-overexpression plants. Both genes work downstream ERF1 in *Arabidopsis* [7]. Therefore, *GhERF12* may activate constitutive ethylene response in the transgenic *Arabidopsis*, and as a result, plant growth is affected.

It is validated that ethylene interact with auxin in regulation of root growth [34]. On the other hand, previous studies reported that both ethylene and auxin act independently in the inhibition of root and hypocotyl elongation in light-grown *Arabidopsis* plants [35,36]. Based on analysis of *alh1* (ACC-related long hypocotyl 1) mutant, it was suggested that the ethylene response is mediated by auxin [37]. Activation of ethylene response results in accretion of auxin in roots [14]. In addition, ethylene stimulates auxin biosynthesis and basipetal auxin transport toward the root elongation zone, where it activates a local auxin response, leading to inhibition of cell elongation [15,17]. A recent study reported that light restores the suppressed root growth of *OsERF1* transgenic *Arabidopsis* plants showing constitutive ethylene response [38]. However, our results indicated that the inhibition on root growth of *GhERF12* transgenic plants seems to be not restorable by light irradiation (Figure 4), suggesting that the role of GhERF12 in ethylene response may be slightly deferent from that of OsERF1.

Previous studies revealed that the ability of the ethylene precursor 1-aminocyclopropane-1-carboxylic acid (ACC) to inhibit root cell elongation is significantly enhanced in the presence of auxin. Ethylene-inhibition of root growth requires auxin response in multiple elongation zone tissues [17]. The plant hormone auxin is well known to stimulate growth at very low concentration, whereas to reduce growth at higher concentration [39]. It is demonstrated that auxin has critical influence on elongation of root cells [40]. Partially dominant mutations of *AUX/IAA*, *AXR3/IAA17*, *SHY2/IAA3*, *AXR2/IAA7* and *MSG2/IAA19* in *Arabidopsis* produce auxin-related phenotypes, including restrained root growth, reduced lateral root formation and reduced gravitropism [41–44]. *35S:HAT2* transgenic plants showed the reduced lateral root elongation and reduced auxin sensitivity, compared with wild type [45]. *GH3* products synthesize IAA–amino acid conjugates. Insertion mutations in *GH3.1*, *GH3.2*, *GH3.5*, and *GH3.17* resulted in modestly increased root sensitivity to IAA [46]. Furthermore, the intracellular level of auxin plays a critical role in regulating the ethylene-mediated growth response in *Arabidopsis* roots [17]. Ethylene regulates root growth by both stimulating the auxin biosynthesis and by modulating the auxin transport machinery [15]. In our study, *GhERF12* expression is induced by IAA in cotton (Figure 2) and primary roots of the transgenic plants were shorter than those of wild type when the seedlings grew on MS medium without or with different concentrations of exogenous IAA (Figure 5). Moreover, expression of the genes related to auxin synthesis (such as *HAT2* and *GH3.2*) and signaling pathway (such as *IAA9*, *IAA17*, *IAA19*, *SAUR* and *PIN1*) was up-regulated in the *GhERF12*-overexpression plants. However, expression of *IAA1* and *IAA3* was down-regulated in the transgenic plants (Figure 6). These data suggested that overexpression of *GhERF12* in *Arabidopsis* may activate the constitutive ethylene response that may stimulate auxin biosynthesis and basipetal auxin transport, and consequently, overhigh endogenous auxin level in cells inhibits growth of the transgenic plants, conferring plants the dwarf phenotype. 
